# Biogeography of terrestrial vertebrates and its conservation implications in a transitional region in western Mexico

**DOI:** 10.1371/journal.pone.0267589

**Published:** 2022-08-05

**Authors:** Andrés García, José F. González-Maya, Gerardo Ceballos

**Affiliations:** 1 Estación de Biología Chamela, Instituto de Biología, Universidad Nacional Autónoma de México, La Huerta, Jalisco, México; 2 Proyecto de Conservación de Aguas y Tierras, ProCAT Colombia/Internacional, Bogotá, Colombia; 3 Departamento de Ciencias Ambientales, CBS, Universidad Autónoma Metropolitana Unidad Lerma, Lerma de Villada, Estado de México, México; 4 Instituto de Ecología, Universidad Nacional Autónoma de México, Ciudad de México, México; Zoological Survey of India, INDIA

## Abstract

Conservation biogeography, which applies principles, theories, and analyses of biodiversity distribution patterns to address conservation challenges, can provide valuable insight and guidance to policy making for protection of biodiversity at multiple scales. The temperate and tropical ecosystems of the Nearctic-Neotropical transition in the small western state of Colima, Mexico, support a mosaic of remarkably diverse fauna and flora and provide a rare opportunity to determine spatial distribution patterns of terrestrial vertebrate species, assess human-induced threats, and identify potential conservation strategies. We analyzed the spatial distribution patterns and correlated them with the current land cover and extent of the protected areas. Despite its limited geographic extension, 29% (866) of all vertebrates, and almost a quarter of both endemic and threatened species in Mexico, live in Colima. Our analysis identified clear high-richness concentration sites (i.e., “hotspots”) coincident for all groups and that elevation and both temperate and tropical ecosystems composition exert significant influence on richness patterns. Furthermore, current species´ distribution also showed significant correlation with natural and disturbed landcover. Significant hotspots for all species groups coincided poorly with the limited protected areas in the state (only 3.8%). The current state of natural land cover (less than 16%) in the state, coupled with its remarkable biological importance, highlights the need for further complementary conservation efforts including expansion and creation of new protected areas, significant restoration efforts and other conservation measures to maintain this uniquely biogeographic and biological diverse region of the country.

## Introduction

Conservation biogeography is an ecological approach that recognizes small-scale conservation may not be adequate to protect regional biodiversity. Information on the spatial and temporal characteristics of biodiversity, such as species distribution [1] is critical for the identification of effects of habitat degradation, land use and vegetation on species conservation [2, 3]. Despite the enormous advances on understanding biogeographic and macroecological patterns of biodiversity [4], and its application to conservation [5], still many aspects remain to be explored to better inform conservation decision-making, especially at sub-global scales [6]. Understanding and exploration of these biogeographic patterns are still particularly scarce for tropical countries [7–11]., ironically, those harboring the greatest levels of biodiversity.

Among such countries, Mexico boasts one of the highest biodiversity and endemism rates in the world [12, 13]. The heterogeneous and very complex spatial distribution of the biota, a product of geological processes, unique biodiversity assemblages, and evolutionary processes, have produced a wide range of species, ecosystems, and ecological associations [13, 14]. And among this heterogeneous and biodiversity-rich landscapes, some regions stand out for their singularity and unique evolutionary and biogeographic characteristics [15, 16]. The great variety of landscapes, physical environments and cultures present in Mexico are reflected through its eight regions that group entities with similar characteristics. These regions are the northeast, northwest, west, east, north-central, south-central, southeast, and southwest, among which the west or west-central region stands out for its rich tapestry of biodiversity and endemism, and which has a very high concentration of vertebrate species in general, and endemic species in particular [16–20], which are also threatened [20–22]. The biogeographic and biodiversity uniqueness of the region is attributable to its location within the Mexican Transition Zone; a zone that includes southwestern United States, all of Mexico and a large part of Central America that has its main division located in the Trans-Mexican Volcanic Belt that marks the main transition in the dominance of the Neotropical or Neartic affinity elements [14, 15, 23]. In west-central Mexico, the location of Colima within the Trans-Mexican Volcanic Belt and the Pacific Lowlands, and the proximity with the Sierra Madre del Sur Provinces, has resulted in its great habitat heterogeneity, terrestrial vertebrate species richness and endemism. The complex biogeography and ecosystem diversity is exemplified by the mixture of terrestrial vertebrate species of very different biogeographical provinces such as those typical of the tropical dry forests (e.g., Guerreran Spiny-tailed iguana (*Ctenosaura pectinata*), Orange-breasted bunting (*Passerina leclancherii)*, and Colima Long‐nosed bat (*Musonycteris harrisoni*), the temperate forests of the Trans-Mexican Volcanic Belt (e.g., Mexican Dusky rattlesnake (*Crotalus triseriatus)*, Mexican Short-nosed skink (*Plestiodon indubitus*), Bell’s false brook salamander *(Isthmura bellii)*, and Allen’s cotton rat *(Sigmodon mascotensis*), and the mixed forest of the Sierra Madre del Sur (Boulder spiny lizard (*Sceloporus pyrocephalus*) [13, 24–30].

The terrestrial vertebrate species of west-central Mexico currently faces severe conservation challenges primarily due to habitat loss and fragmentation, modifying land use and vegetation cover that put populations and entire species at risk, and this scenario is likely to worsen because of climate change [31–34]. Even though the threat of biodiversity loss highlights the need to make regional conservation strategies a priority, it has received little attention in this region of Mexico, and specifically Colima, as evidenced in part by some gaps in the ecological knowledge base and appropriate conservation planning [35].

Effective conservation strategies are critical for the protection of biodiversity, and their development and implementation assume an elevated level of urgency in Mexico, a known megadiverse country for being part of a group of nations containing most of the species on Earth and have a high number of endemic species [36], and especially those regions of the country that support an exceptionally high species richness and uniqueness. Conservation efforts must be grounded in data obtained from comprehensive diagnoses of regional biodiversity [37], and such efforts, are still limited for most of Mexico; species list, such as those conducted in the states of Jalisco, Colima and Michoacán [38–40], have provided baseline data and information, but still spatially-explicit analyses are necessary for the development of viable strategies that include sustainable use options; some of these preliminary good examples include Jalisco and Michoacán [41, 42]; for Colima, as in other entities of the Mexican territory, exercises of ecological territorial planning have been carried out that seek to establish policies of use of the territory based on the vocation of land use and the sustainable use of resources. This policy is binding and serves as a guideline in state development policies [43]. Although these earlier studies were a crucial first step for terrestrial vertebrate conservation, they did not clearly define specific actions that needed to be taken to adequately tackle the primary challenges at the appropriate scale.

Given the need for adequate baseline information and understanding of the biogeographic singularity of a unique state such as Colima, the purpose of this study is to identify those areas deserving conservation actions and identify strategies that target the whole range of terrestrial vertebrate species. We focused on the determination of those areas with higher concentration of vertebrate species number, endemism and endangerment and do an analysis of these patterns with respect to large-scale geographic variables such as land use, land cover, ecological territorial planning, habitat heterogeneity, and topography. These analyses can help facilitate the development of effective conservation strategies and complement those currently employed and to test the hypothesis of the importance of habitat heterogeneity for species richness of terrestrial vertebrates [44].

## Methods

### Study area

**Fig 1:**
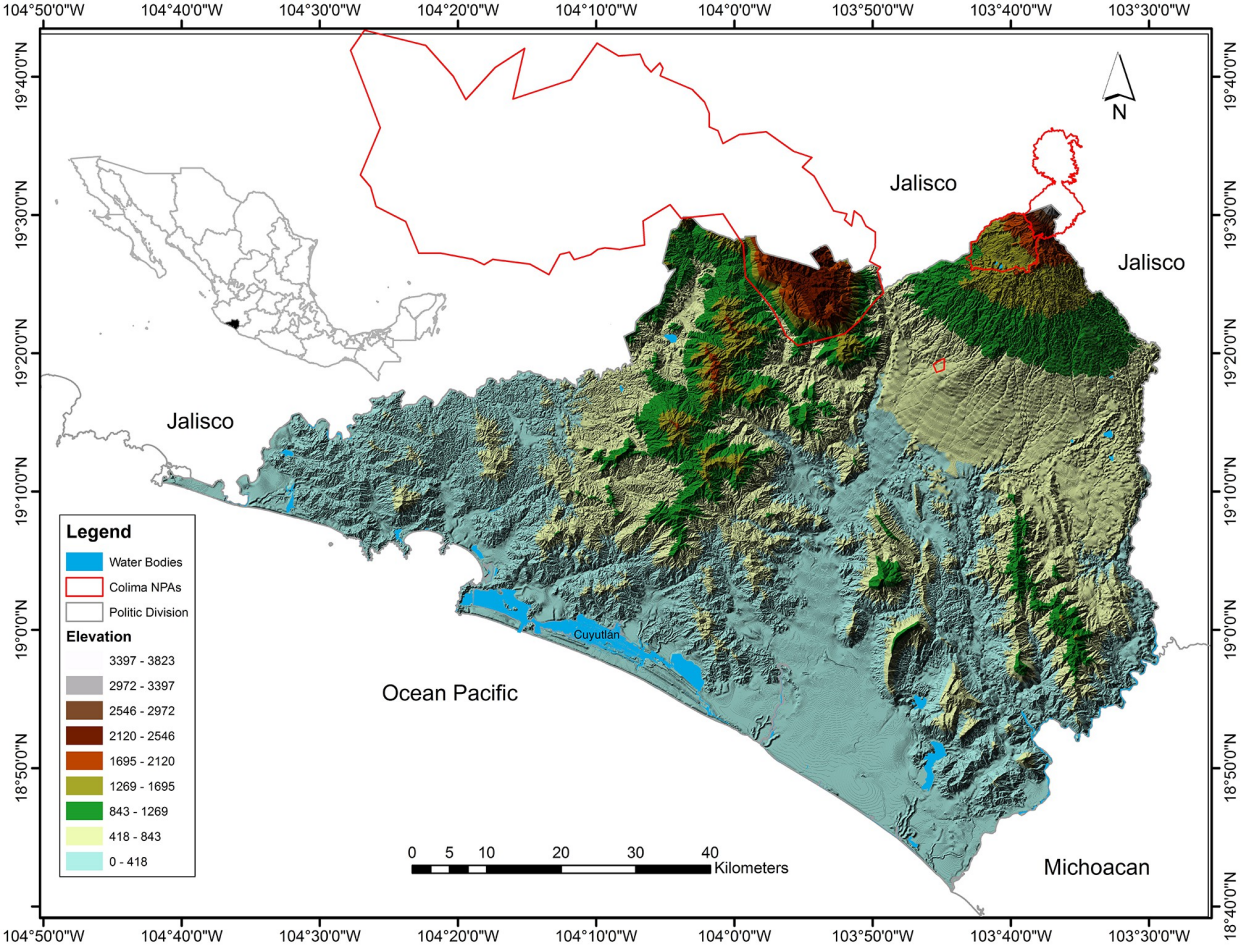
Location of the study area and Terrestrial Natural Protected Areas (NPAs) in Colima State, Mexico [35]. Maps were created by A.G. using ArcGIS ArcGIS 10.2.1. software (https://www.esri.com).

Colima, one of the three smallest political divisions in Mexico and covering 5,542.74 km^2^ (0.3% of the total Mexican territory) is bounded by the 140 km long coastline of the Pacific Ocean to the west and the states of Jalisco to the northwest and northeast and Michoacán to the southeast. Part of the Pacific Lowlands, Trans-Mexican Volcanic Belt and Sierra Madre del Sur Biogeographic Provinces, it lies between 103°29´20” and 104°41’42” W and 18°41’12” and 19° 31’00” N. One of five protected natural areas in Colima, the 148,097.80 km^2^ Revillagigedo Archipelago National Park which includes the islands of Socorro, San Benedicto, Clarion, and Roca Partida, lies 390 km southwest of Baja California and approximately 970 km west of Manzanillo. The other four protected areas, all partially within continental Colima, are El Jabalí Forest Protection and Wildlife Refuge Zone, Las Huertas Natural Resources Protection Area, National Snowy Park of Colima, and the Sierra de Manantlán Biosphere Reserve [38]. The combined area of these sites is 1,513.26 km^2^, but only 212.38 km^2^ (14%) are located within the jurisdiction of Colima which is just 3% of the state territory (Fig 1). The main coastal water bodies in Colima are the Cuyutlán, Alcuzahue, Amela, Potrero Grande, Tecuanillo and Boca de Pascuales lagoons.

This paper focuses only on mainland Colima and therefore does not consider the biota of the Revillagigedo Archipelago or the exclusively marine coastal environment. The major landscape features of Colima consist of two physiographic provinces, four mountain systems and two hydrologic regions. The physiographic provinces, Eje Neovolcánico and Sierra Madre del Sur, can be characterized by their orographic profile: 50% of the area in the eastern sector consists primarily of plains and valleys, 20% of hills with gentle slopes, and 30% hills with steep slopes and 76% of the western portion consists of hills and mountains and 24% valleys and plateaus. Located in the Eje Neovolcánico, Volcan del Fuego de Colima is the highest peak in the state with an elevation of 3,820 masl at the highest point in the volcan de Colima. The four mountain systems include: 1) the highest series of mountains in the state and which are located in the extreme northwest, include Cerro Grande, Sierra Manantlán, Cerro El Peón, Sierra Perote, Cerro la Ocotera, Cerro La Piedra Colorada and Cerro Espumilla Mountains (highest elevation from 1400 to 2420 masl); 2) a series of small mountains (highest elevation is 800 masl) located in the southwest of the state and that run parallel to the coast include hills such as Espinazo del Diablo, El Escorpión, El Tigre, El Aguacate, El Centinela, El Toro (highest elevation from 20 to 748 masl); 3) a series of mountains located between the Armería and El Salado rivers, including Alcomún, San Miguel and San Gabriel Mountains (highest elevation from 1080 to 1400 masl) and; 4) a series located between the El Salado and Naranjo Rivers and which include Picila, Volcancillos, La Palmera, El Camichín and Copales Mountains (highest elevation from 532 to 1066 masl). There are two hydrological regions (RH) in Colima: RH15 embeds the Chacala-Purificación and Cihuatlán or Marabasco Rivers and RH16 the Armería and Coahuayana Rivers.

Although the heterogenous topography modifies local and regional weather patterns, the dominant weather is classified as warm subhumid with 90% of mean annual precipitation (994.9 mm) occurring from June to October, a mean annual temperature of 25°C, and a mean minimum and maximum of 18°C and 30°C respectively. The heterogeneity of weather events and topography account for the wide variety of tropical and temperate forests, wetlands, and other coastal ecosystems which support one of the most biodiverse rich regions in Mexico despite its limited geographical extent. More information about the biotic and abiotic characteristics of Colima can find elsewhere [e.g., 38].

### Vertebrate species and their conservation status

We compiled a comprehensive list of vertebrate species from our field work, specialized literature, and databases maintained by the National Commission for the Understanding and Use of Biodiversity (CONABIO). The IUCN Red List of Threatened Species [45] and the Red List for Mexico [46] were consulted for information on vertebrate species distribution and conservation status, and literature particular for each taxonomic group was used to supplement the information provided. The occurrence reptile and amphibian species in Colima was corroborated in two recent publications [47, 48] and updated reptile and amphibian taxonomy followed [49, 50]. Several papers were consulted for bird and mammals [22, 51–54] as well as specialized databases (Avibase, https://avibase.bsc-eoc.org/checklist.jsp?region=MXcl and the ASM Mammalian Diversity Database (https://mammaldiversity.org) to gather information of their distribution, conservation status and updated taxonomy. The maps used are relatively recent as they come from the continuous review carried out by the IUCN and BirdLife through the consultation of experts; a period of 10 years or less can be considered depending on the species. Although these data are relatively recent, they hardly reflect the current conditions of land use and vegetation type. It is precisely for these reasons that we analyzed the distribution patterns of species richness with respect to land use to assess the potential of threats to biodiversity and define priority areas to be protected. We used these distribution layers because they come from systematic and standardized processes under IUCN standards, thus allow proper comparison given the nature of the construction processes for all groups [11, 55–61]. These expert-opinion maps of species range are constantly updated, are standardized, and include systematic corrections by region and expertise [11, 61]. Furthermore, these polygons have been validated, meet the requirements for proper conservation strategies definition and have been proven useful in multiple previous biogeographic analyses [62–64]. In the results section we report general information regarding all insular (those inhabiting Colima islands) and continental (those inhabiting Continental Colima) species richness and conservation status and then we focus only to continental species, analyze spatial distribution and land use coverage and vegetation.

### Geographical distribution patterns

We used digitalized (shapefiles) geographic distribution maps for Mexican vertebrate species from the BirdLife International and Handbook of the Birds of the World [56] and the Global Mammal, Reptiles and Amphibians Assessments from the IUCN Red List of Threatened Species [45]. Finally, for those few species not included in the assessments but occurring in Colima, we added the polygons of the geographic distribution maps derived from information we gleaned from the literature and databases that provided maps or coordinates of the locality records. All digital maps of Mexican vertebrates were clipped to the boundaries of continental Colima for further analysis and were complemented by the polygons we added for those species not included in the referenced assessments. To create the spatial distribution patterns of species richness, composition, endemism and endangerment of all vertebrate groups, we first create a fishnet of 1 km^2^ (or 100 ha) quadrants within the Colima boundaries and then established their centroids, this resolution was selected as our minimum mapping unit to include some amphibian and rodent species with an extreme restricted geographical distribution [18, 38]; additionally, smaller resolutions will likely increase the spatial correlation between both the range of the smaller species and because the minimum resolution of the environmental covariates used [65, 66]. We enumerated intersecting species distribution polygons to the centroids and estimated species richness, and the number of endemic or endangered species within each grid-cell. The resultant patterns based on expert contour maps and local records were treated as approximations, and we related these patterns with land use, vegetation type, and elevation. We used the list of vertebrate species occurring in each cell to analyze the species similarity between all the 5899 cells covering the study area (34,798,201 comparisons) and estimated the Jaccard index using Phyton (https://www.python.org) to create a histogram of the mean Jaccard index [67] by cell.

### Land use and vegetation

We used vector data sets which contain information at the 1:250,000 scale obtained by photointerpretation from 2014 Landsat 8 satellite images used by the most recent national forest inventory [68] to reclassify the current land use and vegetation into five categories (Supporting Information): 1) conserved, which refers to polygons containing primary vegetation only; 2) disturbed, which refers to polygons with secondary vegetation associated with primary vegetation; 3) transformed, which refers to polygons which have crops or other human-managed vegetation; 4) human settlements; and 5) water bodies.

### Data analysis

Our first treatment in the analysis involved a description of the distribution patterns and given the non-normality of the data, species richness for all groups on each grid-cell were compared using the Spearman correlation test and then plotted. We then estimated the distribution of hotspots across Colima by applying the Getis-Ord Gi* statistic [69] which identifies statistically significant spatial clusters of high values (hotspots) and low values (coldspots) based on a weighted classification (i.e., species richness), indicating where such significant aggregations occur. To conceptualize spatial relationships, we used the Polygon Contiguity function on edge corners which engages all contiguous grid cells sharing a boundary or a node in the estimation and the Manhattan Distance function which estimates distances along axes at right angles [70]. Hotspots were estimated for all (total) vertebrate species and for each vertebrate group and to identify significant aggregations of imperiled species identified on Mexican Red List [46] and endemic to Colima.

We then plotted the values of the four vertebrate groups in each cell to identify their relationship and to determine if one of those groups could be use as surrogate for the others. To determine the influence of environmental variables on species richness for all vertebrates, each group independently and endemic and threatened species [11, 70], we developed a Generalized Linear Model using the Ordinary Least Square (OLS) method. We selected OLS as an appropriate regression model given that it focuses on reducing the total error or the sum of squared residuals; specifically, when dealing with count data (such as species richness) relatively high (usually mean >10) and where variance is not considered small (usually <10), OLS has shown ideal performance and accurate results [71], especially considering its simplicity and the expected linearity of the relationship. We tested the effect and amount of variance explained by four variables accounting for different dimensions of environmental heterogeneity in the state: Elevation, as an altitudinal gradient to investigate how species richness responds to variations in environmental characteristics within small geographical areas [72], Normalized Difference Vegetation Index as a proxy for productivity to predict species richness [73], Potential Evapotranspiration as one of the best climatic correlates of species richness as it is an indicator of climatic and ecological aspects linked to the balance of energy supply and water availability and plant productivity [74] and a Compound Topographic Index as an indicator of geodiversity and environmental heterogeneity promoting species richness [75]. Considering these spatial variables might be correlated or show some collinearity, we tested correlation between each pair by estimating a Pearson correlation coefficient; in case high correlation (Pearson > 0,70) was found for any pair of variables, one would be dropped from candidate OLS models. Further, we tested the spatial autocorrelation of the residuals of the OLS models [76] by estimating the Global Moran´s I statistic, in order to assess if other environmental or biogeographic variables were missing from our models; significant spatial autocorrelation indicates that spatial processes promoting the observed pattern are not random and thus likely other spatial variables can improve the amount of variation explained by the models. These variables have been previously identified as some of the most significant and important environmental determinants of biodiversity, from functional environmental attributes [77, 78] to basic topographic variation [79–82], especially given the heterogeneity of the state, all accounting for spatial attributes usually highly correlated to biodiversity patterns. Given that elevation was a significant driver for all vertebrate groups and is the most prominent and heterogeneous terrain attribute in Colima, we used Elevation as a covariate in a Path Analysis [11] between species richness and land cover; we used this approach to control the effect of elevation by estimating its indirect effect and isolate the potential direct effect of conserved, transformed, and disturbed land use on species richness. Path analyses is a technique ideal to examine the comparative strength of indirect and direct relationship between independent variables, allowing to disentangle such interrelationships between variables [83]; in our case, by using this technique we aimed to better understand how the state of the vegetation could be potentially correlated to the richness of all vertebrate groups.

**Fig 2:**
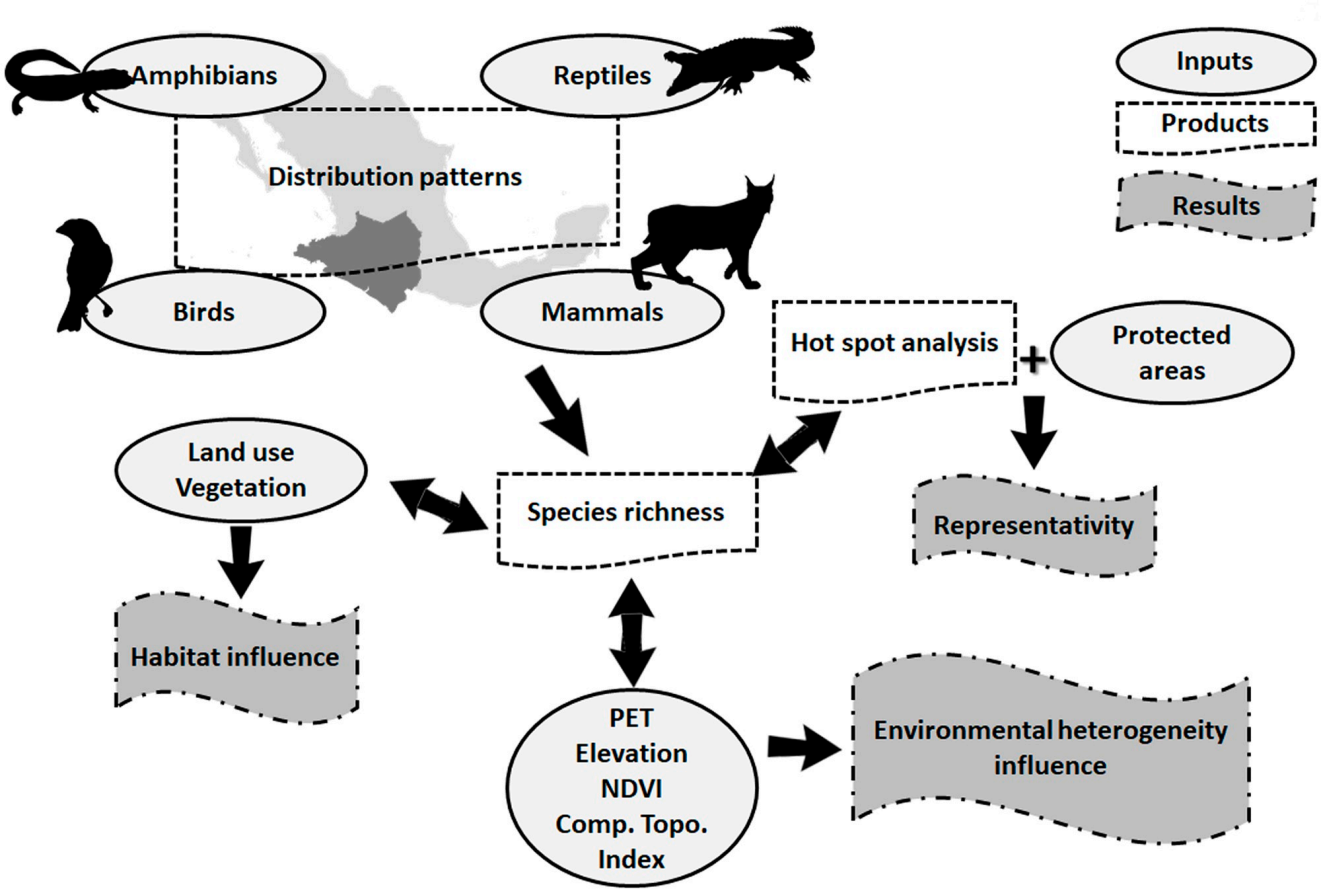
Workflow with the methods and inputs of the manuscript; silhouette imagens taken from PhyloPic (http://phylopic.org).

Finally, we made an overlay species richness and significant hot spot data with the most recent map version of protected areas [84] to determine how well both are represented in protected areas and those areas with high priority for conservation determined by the ecological territorial planning done by the Colima government. This ecological and territorial planning model define, for each environmental management unit (UGA in Spanish), the policies, guidelines and criteria of ecological regulation based on the results of the analytical processes, based on criteria of ecological regulation of the territory defined in the plan of state development and discussion with social actors, the four policies are: protection, conservation, restoration and sustainable use for the entire region; the zoning model of the territory is made up of a series of UGAs [43]. All spatial analyses were performed on ArcGIS and all statistical analyses in R (statistical computing) [85]. Below we present a workflow with the methods and inputs of the manuscript for a better understanding (Fig 2).

## Results

### Species richness and endemism

Colima, home to 867 species including 42 amphibians, 122 reptiles, 545 birds and 158 mammals, has almost one-third of all vertebrate and mammal species, nearly half of the bird and Mexican bat species, and about one-tenth of the reptile and amphibian species in Mexico (Table 1, Supporting Information). About one-fifth of the Colima vertebrate and mammalian species are endemic to Mexico, including more than half of herpetofauna species (especially amphibians) and one-tenth of the bird species (Table 2); there are six species and ten subspecies endemics to Colima (Supporting Information). Since about 10% of the world’s total number of vertebrate species live in Mexico, and the small species-rich state accounts for approximately 3% of world’s total, Colima has an outsized role in contribution to Mexico’s top ranking among the seventeen countries in the world with the highest number species of mammals (third), birds (eleventh), reptiles (second) and amphibians (fifth).

**Table 1:** Vertebrate species in Colima and contribution to Mexican species richness.

Taxa	Orders	Families	Genera	Species	Spp Mex	% Mexico
Amphibia	3	11	22	42	376	11
Reptiles	3	24	73	122	864	14
Aves	25	78	297	545	1,150	47
Mammalia	11	27	99	158	564	28
Vertebrates	42	140	491	867	2,954	29

**Table 2:** Conservation status of vertebrate species in Colima, Mexico; NOM059 (Mexican red list), IUCN, CITES; (% = Colima species with respect to Mexican species).

Taxa	Endemics	NOM059	IUCN	CITES
Amphibia	28 (66.7%)	11 (26.2%)	7 (16.7%)	0 (0%)
Reptiles	70 (57.4%)	59 (48.4%)	12 (9.8%)	5 (4.1%)
Aves	54 (9.4%)	90 (16.5%)	45 (8.3%)	69 (12.7%)
Mammalia	32 (20.8%)	38 (24.1%)	19 (12%)	23 (14.6%)
Vertebrates	184 (21.1%)	198 (22.8%)	83 (9.6%)	99 (11.4%)

### Conservation status

Human activities have severely impacted the natural ecosystems throughout Mexico and imperiled a large proportion of vertebrate species [19, 86, 87], including about 23% of all Colima vertebrates and particularly almost half of the reptilian, a quarter of the amphibian and mammalian and one sixth of the avian species [45]. While almost 10% of all vertebrates and reptilian, about 17% of the amphibian, 12% of the mammalian and 8% of the avian species are listed by IUCN as threatened. None of the Colima amphibians are listed by Convention on International Trade in Endangered Species (CITES) but about 11% of all vertebrates, about 15% of the mammalian, 13% of the avian and 4% of the reptilian species are listed (Table 2). Three recently described frog species, *Eleutherodactylus colimotl*, *E*. *grunwaldi* and *E*. *manantlanensis*, are endemic to Colima only.

### Geographical patterns of distribution of species richness, endemism, and endangerment

**Fig 3:**
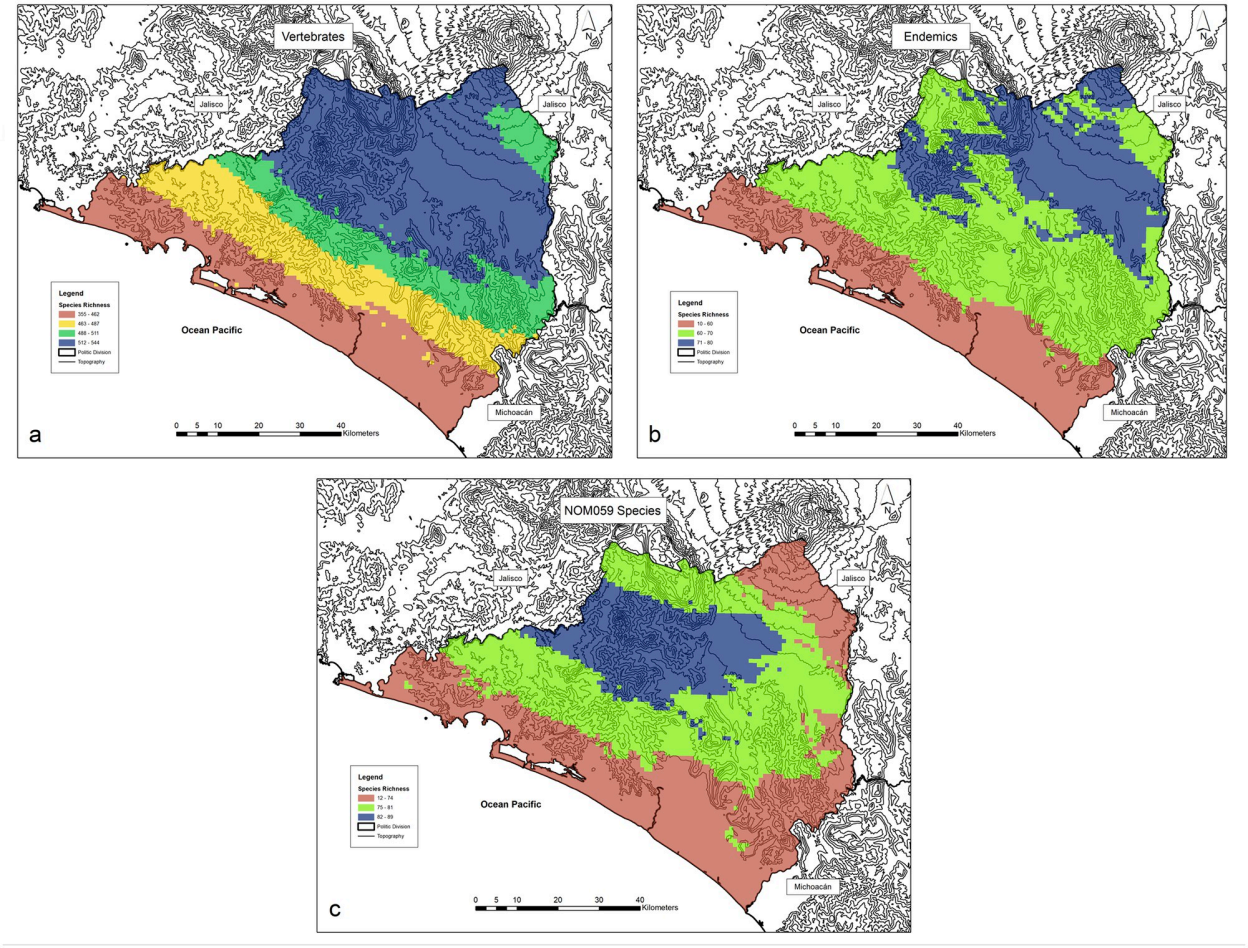
Spatial distribution patterns of (a) vertebrate total species richness, (b) endemic species and (c) threatened species for Colima, Mexico. Note the higher concentration of species in the central and northern regions of the state and the clear gradient for total vertebrate richness. Maps were created by A.G. using ArcGIS 10.2.1. software (https://www.esri.com).

While a large portion of Colima is species rich (Fig 3a), there is a marked spatial distribution pattern of vertebrates in Colima with numbers increasing from 355 to 462 species near the coast to 512 to 544 toward the northern and northwestern regions of the state except for the northeastern tip where the number of species range from 488 to 511. The pattern for the endemic species clearly suggests that the northern sector of Colima is richer than the southern sector. Remarkably, there are at least 50 to 80 endemic vertebrate species in any locality throughout Colima (Fig 3b), and similarly, there are between 59 and 89 threatened species in any region throughout the state (Fig 3c). The histogram of the mean Jaccard index by cell for all the 34,798,201 comparisons of species composition between all cells shows than more than 80% indexes values are in the range of 0.10 to 0.30 (Supporting Information).

**Fig 4:**
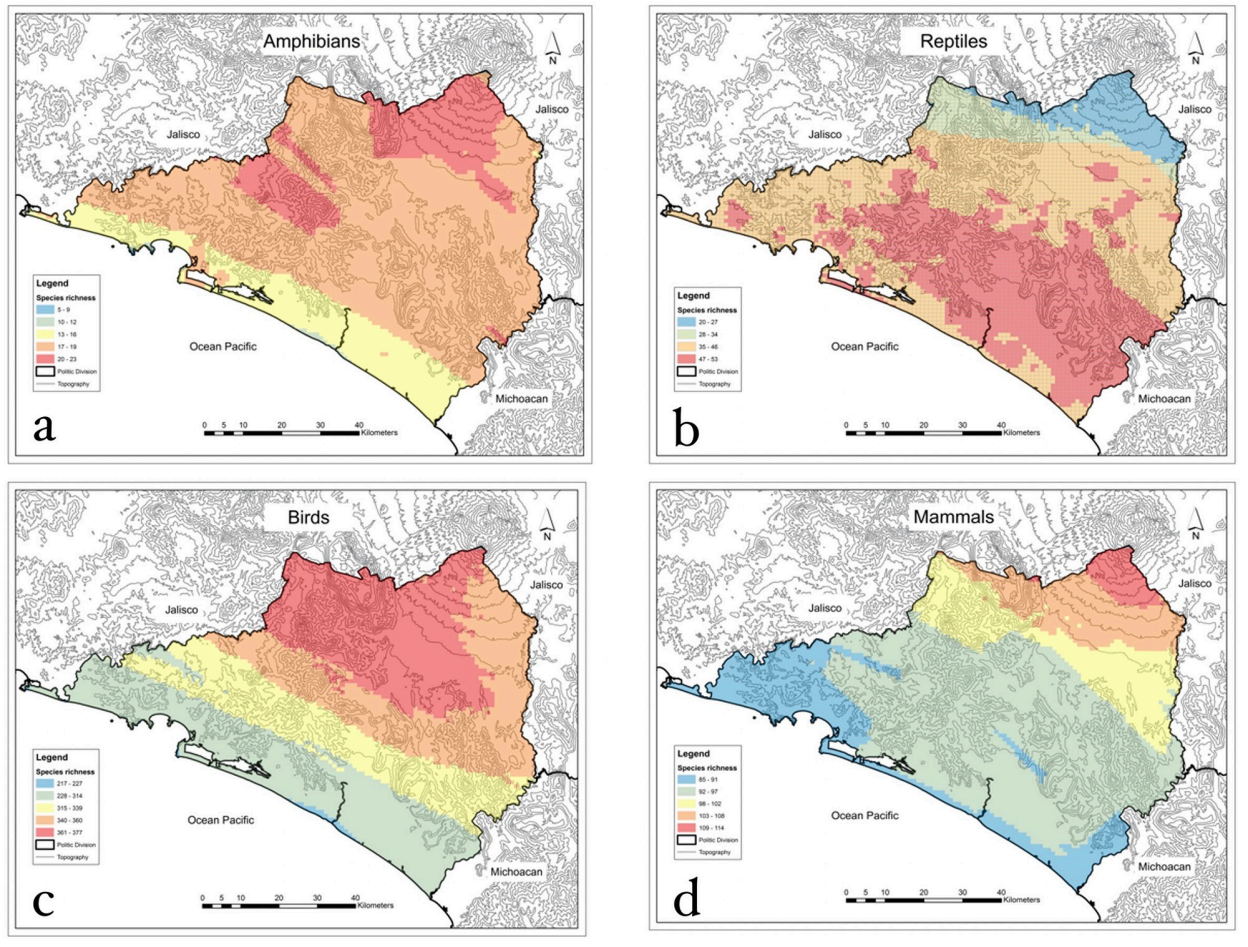
Spatial distribution patterns of species richness by taxonomic group: a) amphibians; b) reptiles; c) birds; d) mammals. Maps were created by A.G. using ArcGIS 10.2.1. software (https://www.esri.com).

**Fig 5:**
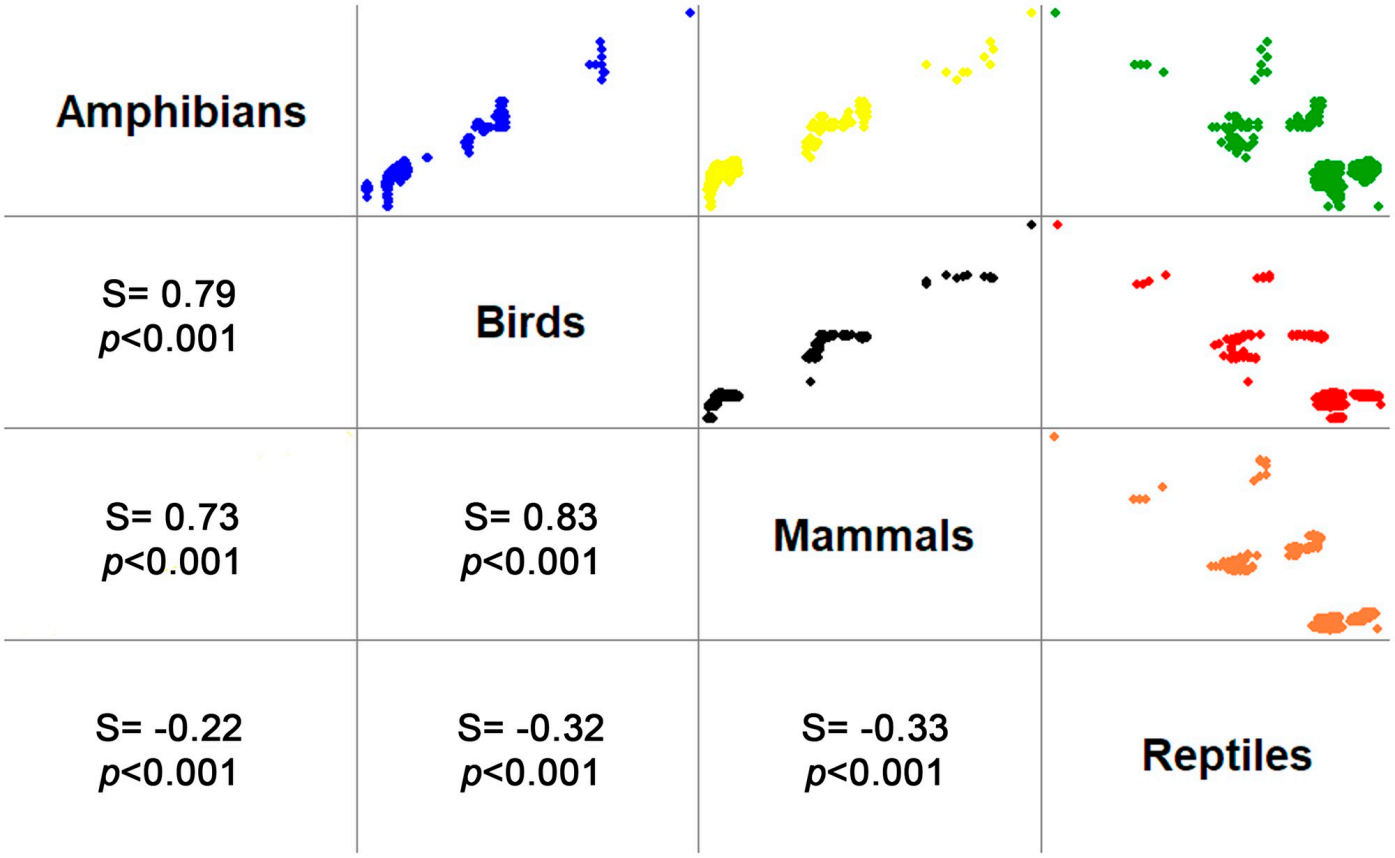
Spearman correlation coefficients (S) and *p-value* (under the diagonal) and scatterplot (over the diagonal) for crossed species richness among groups in Colima, Mexico. Note the high coincidence (trend) between richness values for all groups, and the opposite trend for reptiles in contrast to the other groups.

Spatial distribution of species richness differs by taxonomic group (Fig 4) as exemplified by the contrasting pattern of amphibians and reptiles. There are more amphibian species in northern and eastern Colima in contrast to the coastal areas (Fig 4a) whereas the largest number of reptile species live in coastal areas (Fig 4b). Both the bird (Fig 4c) and mammal (Fig 4d) species were most numerous in the northern half of the state, with the northwestern region particularly favorable for birds. Species richness distribution for the four groups was highly coincident (and related) across the state, with correlation coefficients greater than 0.70 between amphibians, birds, and mammals; reptiles showed the opposite pattern (Fig 5).

**Fig 6:**
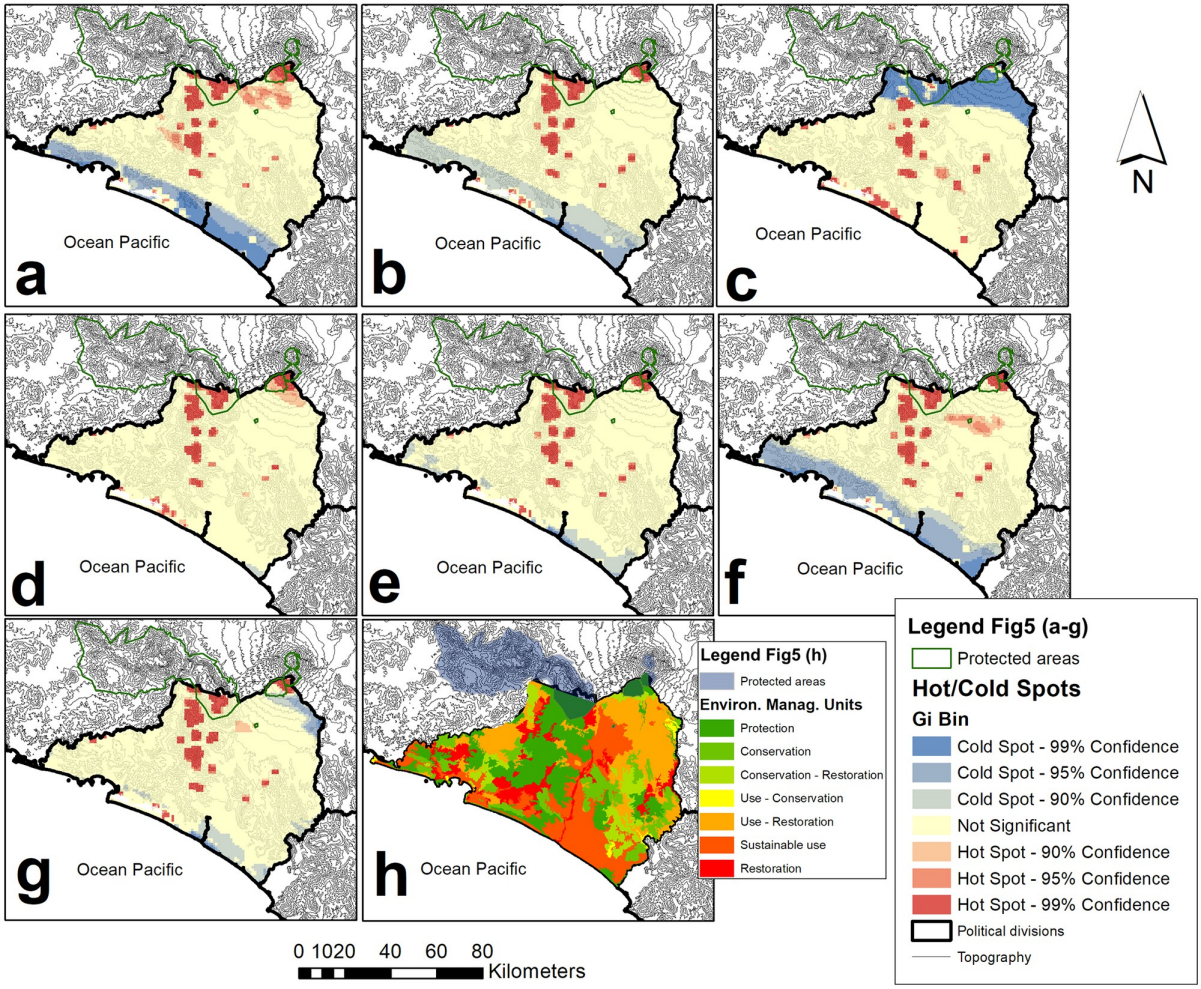
Statistically significant hot spots identified for: (a) amphibians; b) birds; c) reptiles; d) mammals; e) all vertebrate species; f) endemic species; g) threatened species in Colima, Mexico; and h) Environmental Management Units determined by the ecological territorial ordering for Colima. Maps were created by J.F.G.M. using ArcGIS 10.2.1. software (https://www.esri.com).

A total of 744 grid cells were identified as hotspots for all taxon groups, total vertebrates, endemic species, and threatened species (Fig 6), and the spatial patterns were similar for all groups, with a majority associated with the same grid cells (Fig 6). Except for reptiles, there is a spatial concordance of the Terrestrial Natural Protected Areas (NPAs) in northern Colima with the locations of some of the hotspots identified for the other taxon groups. Except for reptiles, there is a spatial concordance of the Terrestrial Natural Protected Areas (NPAs) in northern Colima with the locations of some of the hotspots identified for the other taxon groups (see below). Interestingly, the areas with a high priority for conservation determined by the ecological territorial ordering for Colima has a considerable spatial coincidence with the hotspots determined by our study. Some cold spots, or areas of low clustered species richness, were identified especially towards the coast for all groups but reptiles that showed such spots for the mountain areas; results coincident with the overall distribution of the richness and which is tested later through linear regression techniques (see below).

Regression models between species richness for all vertebrates in general, each group, as well as the endemic and threatened species revealed a significant influence of all variables. No pair of variables showed considerable correlation (Pearson coefficient for Elev vs NDVI = 0,39; Elev vs PET = 0,12; Elev vs CTI = -0,21; NDVI vs PET = 0,54; NDVI vs CTI = 0,039; PET vs CTI = 0,32), therefore we retained all variables for building potential candidate models. The relationship between the different groups of species and each variable varied in terms of intercept, specific influence of each variable (measured by the corresponding coefficient), and especially, the direction of such influence. For instance, Elevation has a negative influence on reptiles, while Potential Evapotranspiration has a negative effect on mammals and birds but not relation with endemic species. Other cases of a lack of relation were found with the Normalized Difference Vegetation Index in amphibians or for the Topographic index with threatened species (Table 3). Total vertebrate richness is significantly influenced by the four assessed variables (R^2^ = 0.45), while threatened species showed the lowest variation explained (R^2^ = 0.11; Table 3). Both residuals map and response curves are included in Supplementary materials (Supporting Information).

**Table 3:** Ordinary least squares regression models parameters and variation explained (R2) by topographic, vegetation and environmental variables as regressor for all vertebrates and for endemic and threatened species richness in Colima, Mexico. Spatial correlation test: M = Moran’s index, z = z-score.

Model	Variable	Coefficient	Standard Error	t	p	R2	M	z	p
All vertebrates	Intercept	463,03	1,28	193,76	<0.001	0,453	0,45	48,07	<0,001
	ELEV	0,05	0,00	45,51	<0.001				
	NDVI	0,00	0,00	-13,91	<0.001				
	PET	0,02	0,00	12,36	<0.001				
	CTI	0,01	0,00	3,60	<0.001				
Amphibians	Intercept	16,66	0,32	52,79	<0.001	0,266	0,50	52,96	<0,001
	ELEV	0,00	0,00	21,44	<0.001				
	PET	0,00	0,00	-3,50	<0.001				
	CTI	0,00	0,00	3,62	<0.001				
Birds	Intercept	334,62	5,65	59,20	<0.001	0,215	0,46	49,40	<0,001
	ELEV	0,08	0,00	19,81	<0.001				
	NDVI	0,00	0,00	-3,36	<0.001				
	PET	-0,01	0,00	-3,15	<0.001				
	CTI	0,01	0,00	3,88	<0.001				
Mammals	Intercept	99,46	1,59	62,41	<0.001	0,194	0,46	48,72	<0,001
	ELEV	0,02	0,00	17,52	<0.001				
	NDVI	0,00	0,00	-2,44	0.01				
	PET	-0,01	0,00	-6,60	<0.001				
	CTI	0,01	0,00	5,31	<0.001				
Reptiles	Intercept	42,90	0,60	71,23	<0.001	0,124	0,61	64,44	<0,001
	ELEV	-0,01	0,00	-18,98	<0.001				
	NDVI	0,00	0,00	6,23	<0.001				
	PET	0,00	0,00	5,50	<0.001				
	CTI	0,00	0,00	-5,40	<0.001				
Endemic	Intercept	61,56	0,96	64,19	<0.001	0,252	0,50	53,31	<0,001
	ELEV	0,02	0,00	22,10	<0.001				
	NDVI	0,00	0,00	-5,60	<0.001				
	CTI	0,00	0,00	4,13	<0.001				
Threatened	Intercept	74,92	1,15	65,14	<0.001	0,111	0,50	53,04	<0,001
	ELEV	0,01	0,00	13,27	<0.001				
	NDVI	0,00	0,00	2,72	0.01				
	PET	0,00	0,00	-3,59	<0.001				

**Fig 7:**
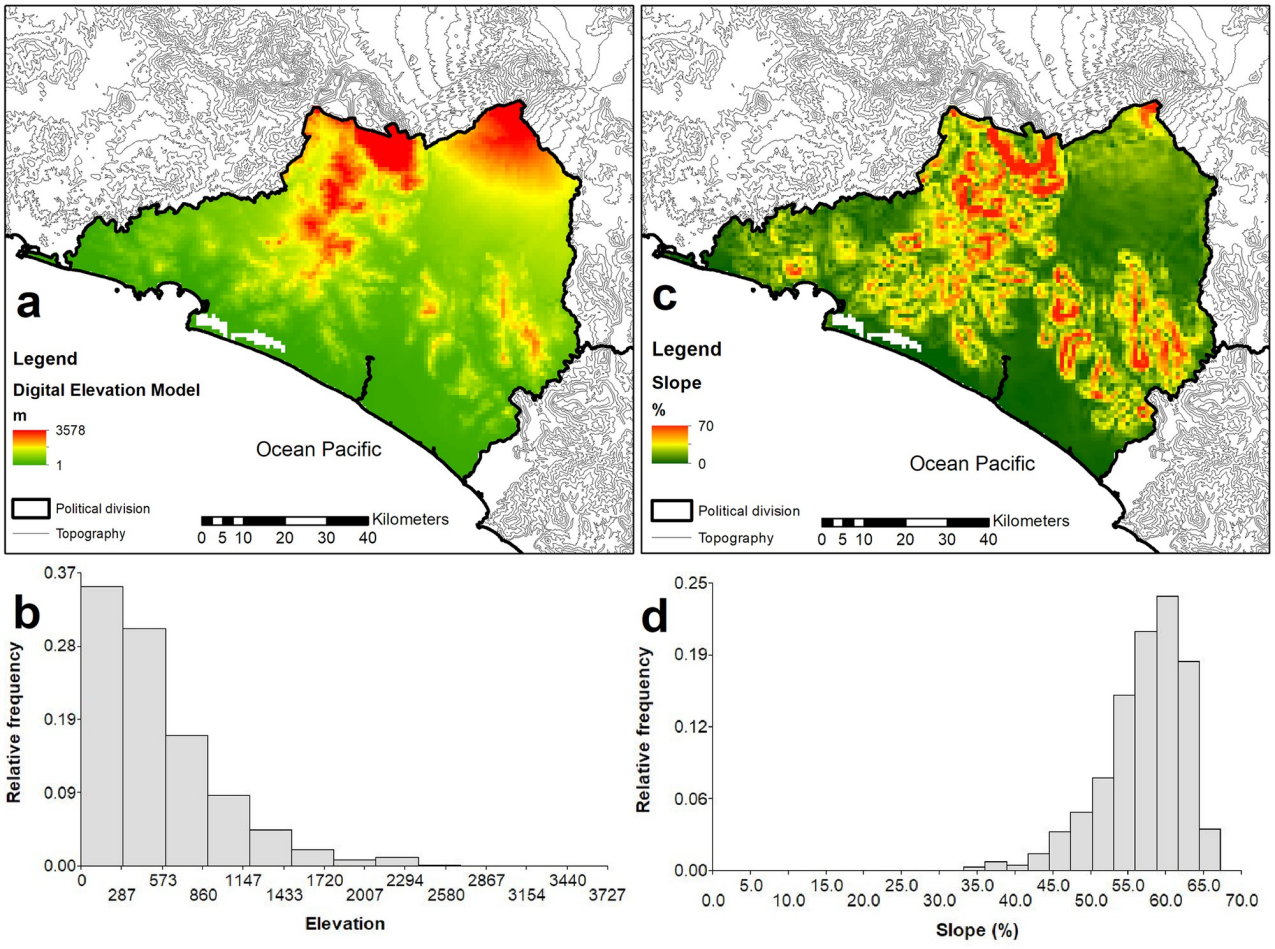
Histogram and spatial distribution of elevation and gradient (percent of slope) across Colima, Mexico. Maps were created by J.F.G.M. using ArcGIS 10.2.1. software (https://www.esri.com).

The topography of Colima is characterized by a steady and generally gradual increase in elevation from the coast to the north and northwest and a significantly more dramatic rise in in the eastern part of the state culminating in the 3,827-meter Volcán de Colima which sits on the border with the neighboring state of Jalisco. Most of the elevation of the state is below 573 masl (mean elevation 511 m, range 0–3,827 m, and a very high standard deviation of 457.71 m) while a significant proportion of the state has a slope greater than 60% (Fig 7). Such topographic variation is also reflected on the very large variation for other environmental variables such as vegetation variation, evapotranspiration and topographic variation, the combination of such heterogeneous conditions is well reflected on its effect on all vertebrate groups assessed (Table 3), also reflecting on its diversity, but indicating potentially other biogeographic factors remain to be explored. Evidence from the potential effect of other biogeographic factors is also supported by some clustering of the residuals for all groups (Table 3).

### Land use and vegetation

**Fig 8:**
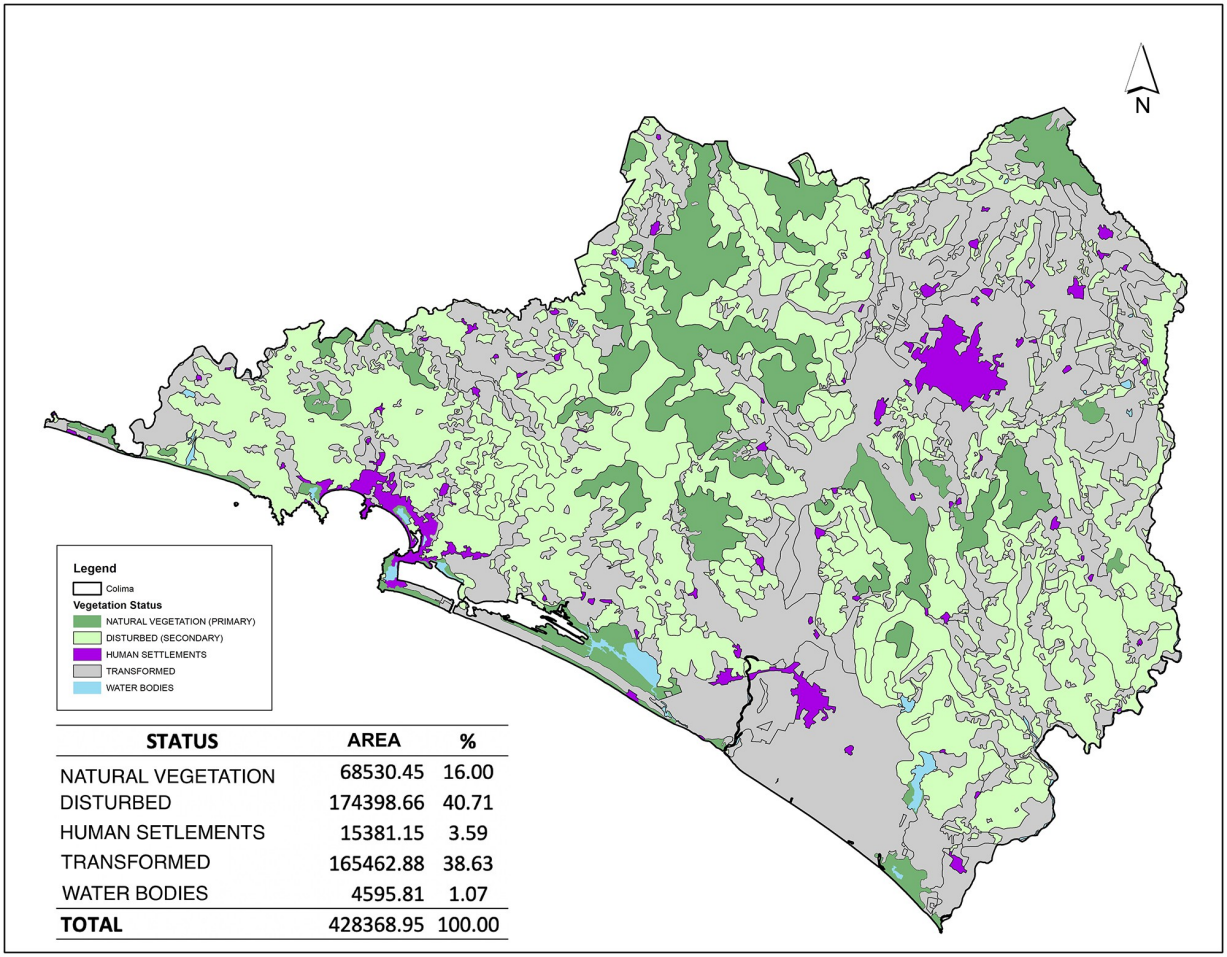
Land use and vegetation. Map were created by A.G. ArcGIS 10.2.1. software (https://www.esri.com).

Our analysis revealed most (83%) of the landscape in Colima has been modified (including 41% classified as disturbed, with secondary growth, and 39% as transformed or agricultural and only 16% was covered by natural vegetation, with primary growth) (Fig 8). Natural vegetation includes fourteen types, six associated with temperate and four each with tropical and coastal ecosystems. Remnants of the natural vegetation are concentrated primarily in the vicinity of Volcan del Fuego, Manantlán-Sierra Perote, and other nearby mountains; the mountainous regions between the Armería and El Salado Rivers such as Alcomún, San Miguel and San Gabriel Mountains; and the upper limits of Cerro Grande, part of the Manantlán Biosphere Reserve, and the El Jabalí and Snowy of Colima NPAs. The areal portion of this natural vegetation includes tropical dry seasonal forest (25.4%), oak forest (23.6%), tropical semideciduous forest (17.4%) and pine-oak forest (9.4%) and accounting for 76% of all-natural vegetation still present at Colima.

**Fig 9:**
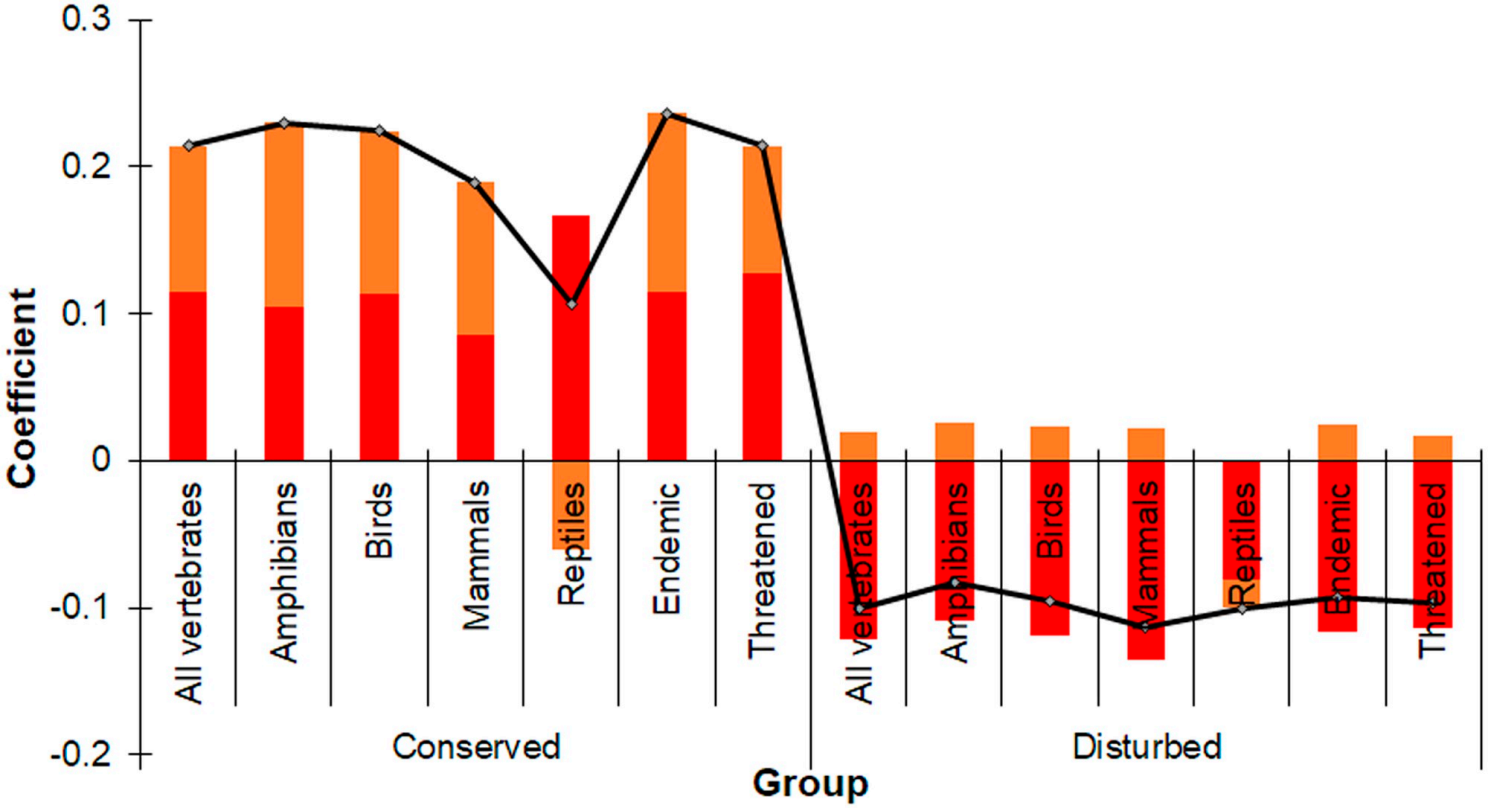
Path analyses of the total correlation coefficients (black line) composed of direct effects of vegetation (red bars) and the indirect effects of elevation (orange bars) in Colima, Mexico. The graph indicated the degree of correlation with land cover once the expected correlation with elevation is explained; positive values indicate positive correlation and negative values negative correlation.

The direct and indirect relationship between land use and species richness (path analysis), once accounting with the effect of elevation, indicated a significant correlation between natural vegetation and all taxa (p<0.001) and between disturbed land and taxon groups and conservation status (all vertebrates: p = 0.0049, amphibians: p = 0.0208, birds: p = 0.0080, mammals: p = 0.0017, reptiles: p = 0.0054, endemic species: p = 0.0098, threatened: p = 0.0072). However, there was no significant correlation between transformed areas and any taxon groups (all vertebrates: p = 0.4168; amphibians: p = 0.2022; birds: p = 0.3471; mammals: p = 0.4658; reptiles: p = 0.6288) and conservation status (endemic species: p = 0.4017; threatened: p = 0.2690). These results suggest there is a direct correlation between species richness and land use and that elevation has an indirect effect. The strongest correlation occurred between natural vegetation and endemic species (0.236), while the weakest correlation was with reptiles, which interestingly had the strongest positive correlation with natural vegetation but a strong negative relationship with elevation (Fig 9). In contrast, species richness for all assessed groups was negatively correlated with disturbed areas, with the largest coefficients for mammals (-0.113) and the smallest for amphibians (-0.083; Fig 8). The indirect effects of elevation on species richness in disturbed areas was positively correlated for all the groups assessed except for reptiles (Fig 9). Finally, as can be seen in Fig 8, there are few human settlements of considerable extension in the state of Colima. When comparing this figure with Fig 6, none of the hotspots correspond to any of these settlements and they do have a spatial correspondence with most of the primary vegetation polygons.

**Fig 10:**
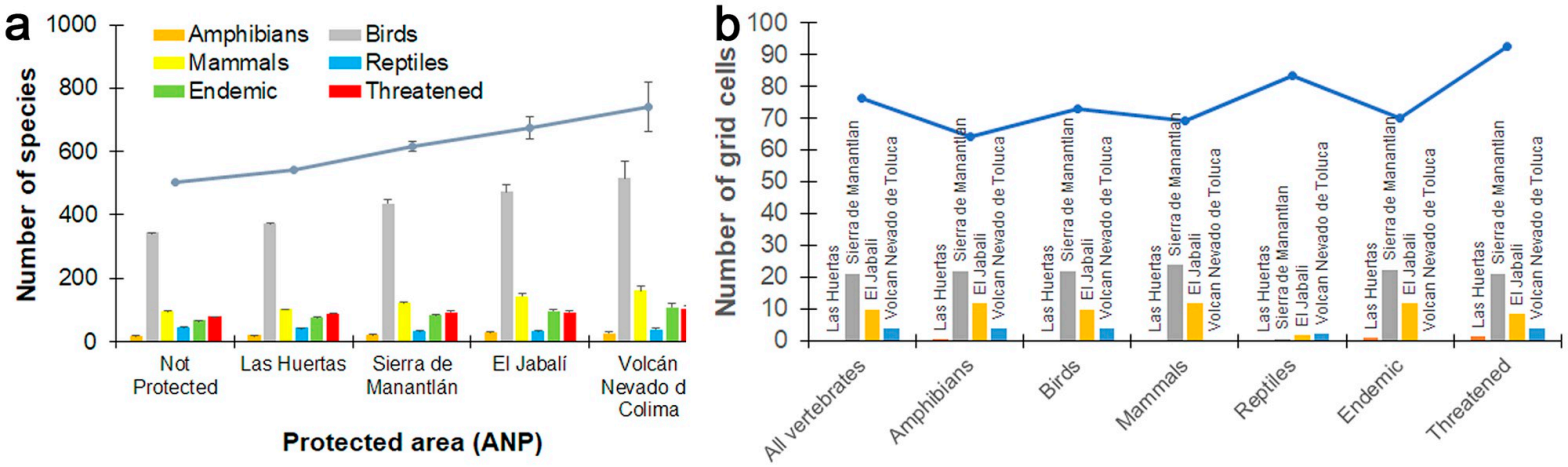
a) Mean number (±SE) of species included in protected and not protected grid-cells and b) number of significant (90, 95 and 99% confidence) hotspot grid-cells included in protected and unprotected areas in Colima, Mexico. Color code (a) all vertebrates: Blue line; amphibians: Orange bar; birds: Gray bar; mammals: Yellow bar; reptiles: Blue bar; endemic species: Green bar; and threatened species: Red bar. The blue line in (b) indicates total number of grid cells in each group located in areas with no official protection status.

Finally, our analyses showed that only 281 grid cells (5% of the total) were included in any protected area, but their location is highly significant in terms of species richness. When all vertebrates (Fig 10a), and each taxon and conservation status metric (endemic, threatened) (Fig 10b) are considered, the mean number of species is larger in the grid cells representing protected areas than in the remaining (and much more numerous) grid cells representing unprotected areas. Among the four protected areas, the National Snowy Park of Colima has the largest mean number of species. Only 28% of the hot spot grid cells were at least partially included in a protected area and threatened species (in hot spots) predominantly showed up in grid cells that represented unprotected areas. The Sierra de Manantlán Biosphere Reserve, a protected area, was one of the areas with the largest number of grid cells identified as significant hotspots, whereas the Las Huertas Natural Resources Protection Area, another protected area, had the lowest number of hot spot grid cells (Fig 10b).

## Discussion

In this work, as we expected, the small state of Colima contains a high degree of species richness and endemism. This rich biodiversity could be related to the geographical position, environmental heterogeneity, and geological history of this territory. There are several studies that detail the importance of this region due to its geological past, location, considerable environmental heterogeneity, and biodiversity [14, 16, 17, 21, 22, 29, 35, 48, 88–90]. The small area of the state accounts for a large proportion of Mexican vertebrate species richness, endemism, and endangerment: approximately one-third of the total vertebrate species in Mexico, 16% of the 1,127 Mexican endemic species, seventeen vertebrate species endemic only to this Colima, and 15% of the 1,320 vertebrate species protected by the Mexican law [22, 46–48, 51–53]. Colima is an extremely important hotspot for some groups such as Mexican bats and birds. Half of the Mexican bat [46] and Mexican bird species [53] are found in Colima. The area plays an outsized role in bird migration; about 40% of the Colima bird species are migrants [91]. It was not possible for us to determine the Neotropical or Neartic affinity for each of the species present in the state of Colima because this type of revision has fallen into disuse since the taxonomy of the groups is in continuous revision and rearrangement, complicating this kind of analyzes. We suggest that the biogeographic and biodiversity uniqueness of the region is attributable to its location within the Mexican Transition Zone, the location of Colima within the Trans-Mexican Volcanic Belt and the Pacific Lowlands, and the proximity with the Sierra Madre del Sur, which gives to the small state Colima, an outsized role in contributing to Mexico’s top ranking among the seventeen megadiverse countries in the world [36].

Our results indicate that vertebrate species richness in Colima increases gradually from the coast to higher elevations in northern and northwestern Colima. While bird, mammal and amphibian species richness followed similar patterns, it was the opposite for reptiles. Taking into consideration ecological characteristics, this similarity in spatial patterns (except for reptiles) indicate that members of one group may be used as an indicator of the distribution of the other groups. This “substitution” may facilitate systematic conservation planning and establishment of protected areas [92, 93]. Furthermore, our results indicate a case of cross-taxon congruence that have been reported at different geographical scale and for different taxa, and where the factors involved depended on of the spatial extent and the grain size under analysis and concluded that climatic predictors of species richness are stronger at the regional scale [94, 95]. The mean Jaccard index by cell that showed a clear dissimilarity of species composition between all cells along the study area suggesting a high beta diversity across the landscape [96, 97], a topic for further analysis in another paper.

Understanding the strong relationship between geography and species characteristics (i.e., spatial orientation, richness, endemism, and endangerment) at multiple scales is a critical component of biodiversity conservation. For instance, the distribution pattern for the herpetofauna species richness along the Pacific lowlands and adjacent interior valleys of Mexico is similar to those for the endemic and endangered species probably because the high endemism and endangerment (30% across the region) is driving the species richness pattern [22]. This spatial relationship is, however, scale-dependent, being greater at larger scales (e.g., entire biogeographic region) or involves threshold number of endemics [98, 99]. The small geographic size of Colima, the ecological and geographic isolation of the main vegetation type, seasonally dry tropical forests [16, 22], and the uniqueness and isolation of its species are some of the contributing factors that account for the highest concentration of vertebrate species in Mexico, one of the highest rates of endemism in the country, strong patterns of spatial relationships, and ironically contributes to the endangerment of many species.

The extreme biogeographical heterogeneity and biodiversity of Colima, especially in the northern reaches of the state, is probably attributed to its geographical location. Colima is situated within both the Neotropical Realm and Mexican Transition Zone which include the contact zone of the Pacific Lowlands, Trans-Mexican Volcanic Belt and Sierra Madre del Sur Provinces where the Neotropical and Neartic biotas overlap [14, 15, 23]. These geophysical conditions and the region’s climate regime support an extraordinary number of endemic and microendemic species from area or the occurrence of species from distant regions that enrich the fauna of Colima [22, 47, 88–90, 100]. Some examples of microendemic species or those with very limited distribution in northern Colima and whose geographic range extends to other physiographic regions and biogeographical provinces include: *Isthmura bellii* [24]; *Crotalus campbelli*, a new described species from the *C*. *triseriatus* species group [101]; and *Cryptotis alticola* [102]. Central western México is considered an important center of endemism for vertebrate, invertebrate, and plant species [100, 103, 104] and the center of origin for the *Crotalus* and *Sistrurus* genera [105].

The hotspots of all vertebrate groups, endemic and threatened species showed a similar spatial distribution across Colima; however, this pattern was not replicated among some groups for the coldspots. Only 28% of the hotspot grid cells are located within any of Colima’s four NPAs, and they do not represent enough area to protect all the hot spots and other conservation priority areas. These NPAs and the Cuyutlán Ramsar Site (Basin III and IV of Laguna Cuyutlán, not included within the NAPs Mexican system) which protect many different groups of vertebrates, endemics, and threatened species, currently enjoy formal protection because of their exceptional ecological and biodiversity value. For example, the 7,200-ha Laguna Cuyutlán (35 km long and 6 km in its widest point) which encompasses the 4,051 ha Ramsar site, is the largest wetland in a span of 1,150 km along the Pacific Coast [106, 107]. This lagoon represents 90% of wetlands in the state, is the fourth largest coastal wetland in the country, and is characterized by a diversity of flora and wildlife. It provides shelter to at least 160 bird species including 25 waterbird species that nest there and 61 waterbird species that use the lagoon during their non-breeding season. In contrast, the largest number of grid cells depicting threatened species hotspots are in areas that currently lack any formal protection status. We recognize that the determination of critical areas for conservation based solely on the concentration of species richness, endemism and endangerment has some limitations. We use contour maps to determine spatial distribution patterns to compensate for the lack of specific information from field studies that cover the entire surface of Colima. Also, due to the low number of Terrestrial Natural Protected Areas (4) and their reduced geographical extension in Colima (3.8%) we did not focus on analyzing their effectiveness or do any complementary analysis. Instead, as a proof of concept, we simply assessed how cells that represent hotspots are currently covered by the protected areas system. We did not assess any metrics related with effectiveness but provide the baseline for further analyses. Although we acknowledge how other systematic conservation planning approaches could complement our approach, our main purpose was to explore biogeographic patterns, identifying where significant clusters of high species richness occurs and how well represented these unique sites are in the existing protected areas; an approach also used previously for identifying where conservation attention should be focused [108].

The use of multiple environmental variables to understand their influence on diversity patterns has been at the core of biogeographic research [109, 110], and especially to identify areas of conservation importance at the regional [60, 110, 111] and global scale [112–114]. We applied this methodology to determine the spatial distribution of Colima vertebrate species richness, endemism, and endangerment to provide tools to enhance biodiversity conservation in the state. The close relationship of elevation, climate, and vegetation, especially at a regional scale [115], was validated by our linear models which showed a significant positive influence of elevation on the spatial distribution pattern of species richness for all taxonomic vertebrate groups, endemism, and endangerment except reptile species richness (negative). The models also revealed that climate and possibly the historical vegetation cover also influenced species richness, given the strong correlation between land cover and richness values. Given that elevation and potential evapotranspiration were also identified as an important variable for all groups, it is likely climate will also exert an important influence on the distribution of richness. The climate in the largest portion of Colima (86%) is classified as warm sub-humid, in the Sierra de Perote and associated mountains (12.5%), as dry and semi-dry, and in the Cerro Grande and region of the National Snowy Park of Colima (1.5%), as sub-humid. The historical vegetation consisted of tropical forests (83%), temperate forests (13%), mangrove swamps (3%), and cloud forests and other types of vegetation (1%) (SEMARNAT, 2001). The relative uniform conditions of climate, vegetation, and topography in most of the state may explain the spatial distribution of animal species at the smaller scale (larger area) while ecological factors are determinative factors at the larger scale (smaller area). The reduced species richness of reptiles in the northern and regions of Colima that have higher elevations is related to the temperate climate because their distribution is largely determined by the amount of radiant heat [116]. Overall and as predicted by the habitat heterogeneity and species richness hypothesis [44], it is clear environmental heterogeneity is a strong determinant of species diversity, which is not surprising giving the interesting composition of the state with multiple natural regions converging into a relatively small area. Within this environmental heterogeneity, it seems habitat heterogeneity, mostly reflected on vegetation-variables, exert a significant effect over the distribution of all vertebrate groups; such a relationship, together with the direct relationship with habitat disturbance, poses an even more dramatic scenario for vertebrates in the state.

We contend that the strong spatial relationships we identified provides a valuable tool for immediate and longer-term systematic conservation planning by facilitating identification of critical areas throughout the state. Protection of Colima hotspots must be an urgent priority because of the rapid loss of natural vegetation and overall habitat loss in recent years. Currently, more than 80% of the landscape in Colima has been transformed or disturbed to varying degrees and only 16% has been conserved or has natural vegetation. Even though the Colima NPAs protect an important number of species, some of these areas have suffered severe deforestation and their role as refugia has been compromised. A comparison of the historical primary vegetation cover [117] with the most recent forest inventory [61] revealed a loss of at least 84% of the primary vegetation, including 94% of the tropical and 63% of the temperate forests. The relatively few remnants of naturally conserved vegetation in Colima are at the higher elevations (> 700 masl) and show a strong relationship with areas of high species richness. Our research shows a high correlation between the amount of conserved land and species richness for all taxonomic groups and the endemic and threatened species and a significant indirect relationship with elevation. The higher elevation areas, some of which still retain remnants of natural vegetation, coincidentally are the areas with the greatest species richness for all taxonomic groups except for reptiles, which are more numerous at lower elevations. Although this pattern is not surprising [118], it further supports the need to secure the remaining habitats through expansion of the NPAs system to include those hot spots currently vulnerable to disruptive human activities, such as land conversion. The areas with a high priority for conservation determined by the ecological territorial ordering for Colima has a considerable spatial coincidence with the hotspots determined by our study. This supports our findings and suggestions to protect that remaining habitat through the expansion of the NPAs system to include those hotspots, while also embarking on land restoration projects throughout the state to recreate natural connections and corridors, such as one extending from Cerro Grande (Sierra de Manantlán Biosphere Reserve) to the lowlands adjacent to Laguna de Cuyutlán to include this wetland [119], the associated ecosystems and the hotspots determined by this study within the Mexican NPA system to protect them against the effects of the current infrastructure development in the region. Our data, which showed low species richness areas for all groups were coincident with areas that were disturbed but still maintained secondary vegetation and the absence of correlation between severely degraded areas (transformed vegetation) and species richness, supports the need for robust land restoration efforts to conserve biodiversity. These and other conservation other strategies (e.g., financial incentives to protect wildlife) must be pursued to prevent further erosion of Colima’s extraordinary species diversity. Despite the large percentage of Colima’s landscape that has been transformed to meet human needs, the relatively small size of the state may better facilitate robust conservation efforts going forward to protect and enhance the biodiversity and uniqueness of this region in Mexico. The measures to extend protections require improving management of existing protected areas, expansion of existing ANPs and the creation of new ones by securing remnant areas of natural land cover and restoration of disturbed habitats, and implementation of alternative and complementary conservation strategies [120] such as voluntary protection of land, community protected areas, and incentives (such as payment) for environmental services and management units for wildlife protection. Although the previous exercise of territorial ecological planning for the state of Colima coincides in some points with the principles of systematic conservation planning, it did not use the modern methods of this approach. It is the result of a first approximation that supports the importance of the areas of high priority defined by our study, however such ecological territorial planning must be verified by means of adequate modern techniques [121, 122]; such studies should be implemented in different areas and a global scale.

## Supporting information

S1 Fig Jaccard index histogram. (JPG)

S2 Fig Residuals maps. (JPG)

S1 File Vegetation reclassification from INEGI Serie VI for Colima. (DOCX)

S2 File Species checklist and status conservation. (XLSX)

S3 File Response curves for all selected OLS models. (DOCX)
