# Supplementary figures and images for: Biogeography of terrestrial vertebrates and its conservation implications in a transitional region in western Mexico
Source: PLoS One. 2022 Aug 5;17(8):e0267589. doi: 10.1371/journal.pone.0267589 (PMC9355201; doi:10.1371/journal.pone.0267589)

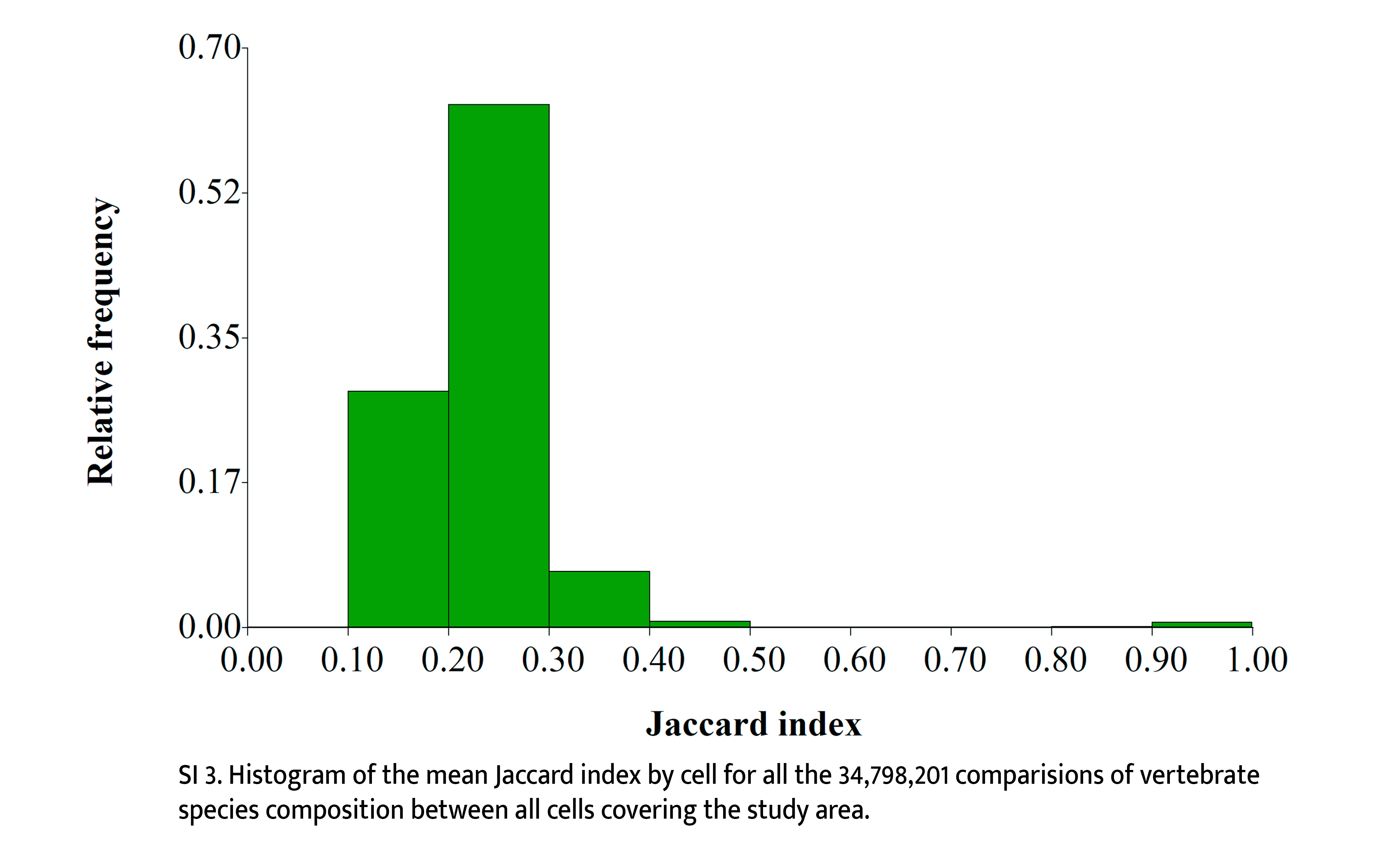

Supplement: S1 Fig — (JPG) [file pone.0267589.s001.jpg]

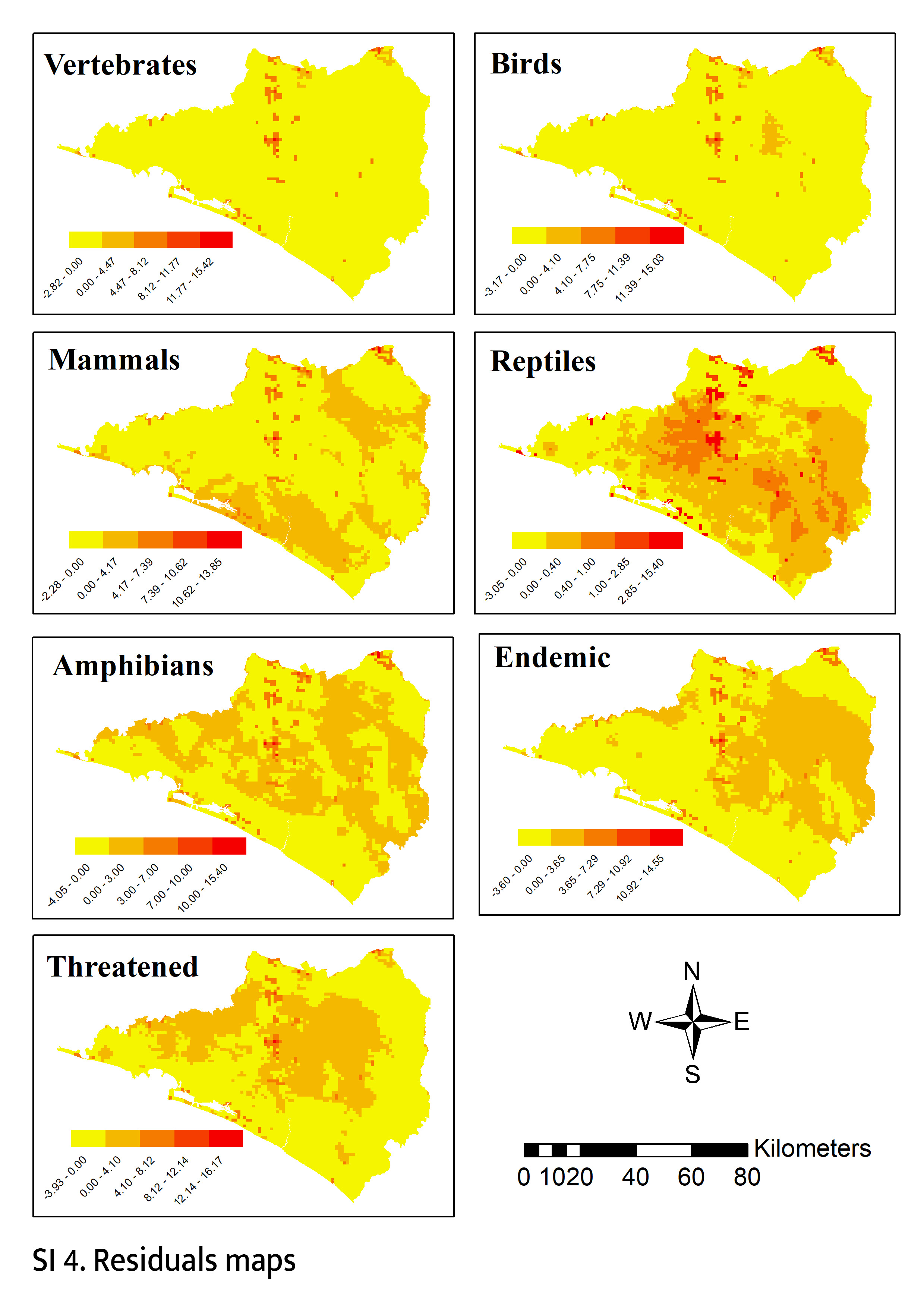

Supplement: S2 Fig — (JPG) [file pone.0267589.s002.jpg]
