# Supplementary material for: Biogeography of terrestrial vertebrates and its conservation implications in a transitional region in western Mexico
Source: PLoS One. 2022 Aug 5;17(8):e0267589. doi: 10.1371/journal.pone.0267589 (PMC9355201; doi:10.1371/journal.pone.0267589)
Supplement: S1 File — (DOCX) [file pone.0267589.s003.docx]

**SI 1. Equivalences of the classification used with respect to the original description of the polygon from INEGI Serie VI for Colima.**

| *CONSERVED* | *68530.45* |
| --- | --- |
| BOSQUE DE ENCINO | 16169.67 |
| BOSQUE DE ENCINO-PINO | 6471.08 |
| BOSQUE DE PINO | 1504.39 |
| BOSQUE DE PINO-ENCINO | 3594.61 |
| BOSQUE MESOFILO DE MONTAÑA | 1184.56 |
| MANGLAR | 2474.11 |
| MEZQUITAL TROPICAL | 324.39 |
| PRADERA DE ALTA MONTAÑA | 691.14 |
| SABANOIDE | 1050.68 |
| SELVA BAJA CADUCIFOLIA | 17406.48 |
| SELVA MEDIANA SUBCADUCIFOLIA | 11923.31 |
| TULAR | 229.98 |
| VEGETACIÓN DE DUNAS COSTERAS | 1462.21 |
| VEGETACIÓN HALÓFILA HIDRÓFILA | 4043.84 |
| *DISTURBED* | *174398.66* |
| BOSQUE CULTIVADO | 68.51 |
| VEGETACIÓN SECUNDARIA ARBÓREA DE BOSQIE DE ENCINO | 3496.64 |
| VEGETACIÓN SECUNDARIA ARBÓREA DE BOSQUE DE ENCINO-PINO | 160.76 |
| VEGETACIÓN SECUNDARIA ARBÓREA DE BOSQUE DE PINO | 190.35 |
| VEGETACIÓN SECUNDARIA ARBÓREA DE BOSQUE DE PINO-ENCINO | 516.85 |
| VEGETACIÓN SECUNDARIA ARBÓREA DE BOSQUE MESÓFILO DE MONTAÑA | 755.9 |
| VEGETACIÓN SECUNDARIA ARBÓREA DE MANGLAR | 422.96 |
| VEGETACIÓN SECUNDARIA ARBÓREA DE SELVA BAJA CADUCIFOLIA | 25798.27 |
| VEGETACIÓN SECUNDARIA ARBÓREA DE SELVA MEDIANA SUBCADUCIFOLIA | 18022.73 |
| VEGETACIÓN SECUNDARIA ARBUSTIVA DE BOSQUE DE ENCINO | 18594.29 |
| VEGETACIÓN SECUNDARIA ARBUSTIVA DE BOSQUE DE ENCINO-PINO | 636.26 |
| VEGETACIÓN SECUNDARIA ARBUSTIVA DE BOSQUE DE PINO | 145.85 |
| VEGETACIÓN SECUNDARIA ARBUSTIVA DE BOSQUE DE PINO-ENCINO | 53.56 |
| VEGETACIÓN SECUNDARIA ARBUSTIVA DE BOSQUE MESËFILO DE MONTAÑA | 503.51 |
| VEGETACIÓN SECUNDARIA ARBUSTIVA DE MANGLAR | 1046.15 |
| VEGETACIÓN SECUNDARIA ARBUSTIVA DE SELVA BAJA CADUCIFOLIA | 73262.86 |
| VEGETACIÓN SECUNDARIA ARBUSTIVA DE SELVA MEDIANA SUBCADUCIFOLIA | 30002.74 |
| VEGETACIÓN SECUNDARIA HERBÁCEA DE SELVA BAJA CADUCIFOLIA | 720.47 |
| *HUMAN SETTLEMENTS* | *15381.15* |
| ASENTAMIENTOS HUMANOS | 15381.15 |
| *TRANSFORMED* | *165462.88* |
| ACUÍCOLA | 277.34 |
| AGRICULTURA DE RIEGO ANUAL | 8428.14 |
| AGRICULTURA DE RIEGO ANUAL Y PERMANENTE | 7834.31 |
| AGRICULTURA DE RIEGO ANUAL Y SEMIPERMANENTE | 1241.32 |
| AGRICULTURA DE RIEGO PERMANENTE | 12608.85 |
| AGRICULTURA DE RIEGO SEMIPERMANENTE | 162 |
| AGRICULTURA DE RIEGO SEMIPERMANENTE Y PERMANENTE | 21506.19 |
| AGRICULTURA DE TEMPORAL ANUAL | 35466.79 |
| AGRICULTURA DE TEMPORAL ANUAL Y PERMANENTE | 5240.22 |
| AGRICULTURA DE TEMPORAL ANUAL Y SEMIPERMANENTE | 15989.08 |
| AGRICULTURA DE TEMPORAL PERMANENTE | 2164.59 |
| AGRICULTURA DE TEMPORAL SEMIPERMANENTE | 469.37 |
| AGRICULTURA DE TEMPORAL SEMIPERMANENTE Y PERMANENTE | 2411.22 |
| DESPROVISTO DE VEGETACIÓN | 1399.9 |
| PASTIZAL CULTIVADO | 45751.71 |
| PASTIZAL INDUCIDO | 4096.07 |
| SIN VEGETACIÓN APARENTE | 415.78 |
| *WATER BODIES* | *4595.81* |
| CUERPO DE AGUA | 4595.81 |
| **TOTAL** | **428368.95** |
