# Supplementary material for: Biogeography of terrestrial vertebrates and its conservation implications in a transitional region in western Mexico
Source: PLoS One. 2022 Aug 5;17(8):e0267589. doi: 10.1371/journal.pone.0267589 (PMC9355201; doi:10.1371/journal.pone.0267589)

SI 5. Response curves for all selected OLS models explaining species richness for A) All vertebrates, B) Amphibians, C) Birds, D) Mammals, E) Reptiles, F) Endemic species and G) threatened species.


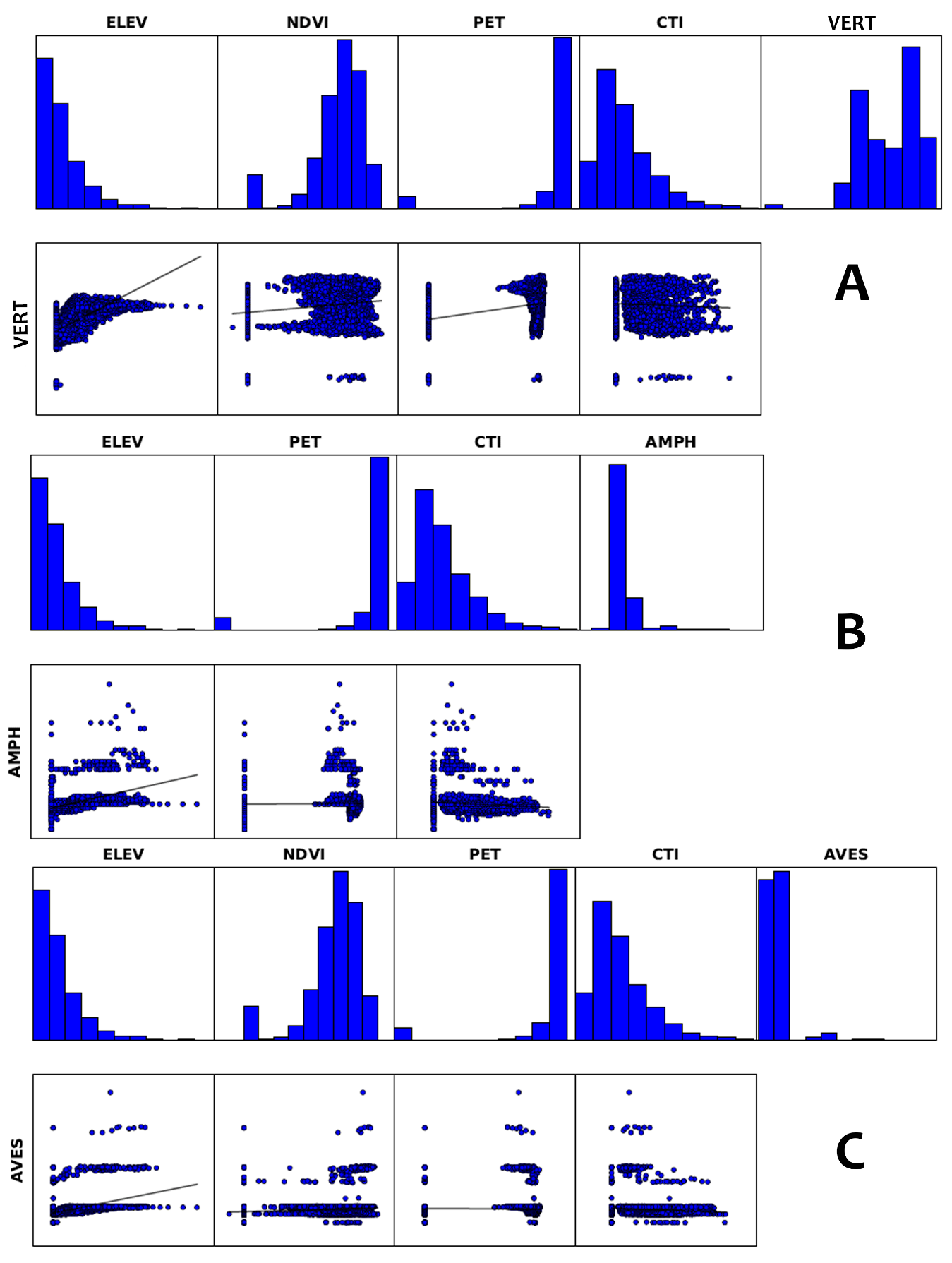


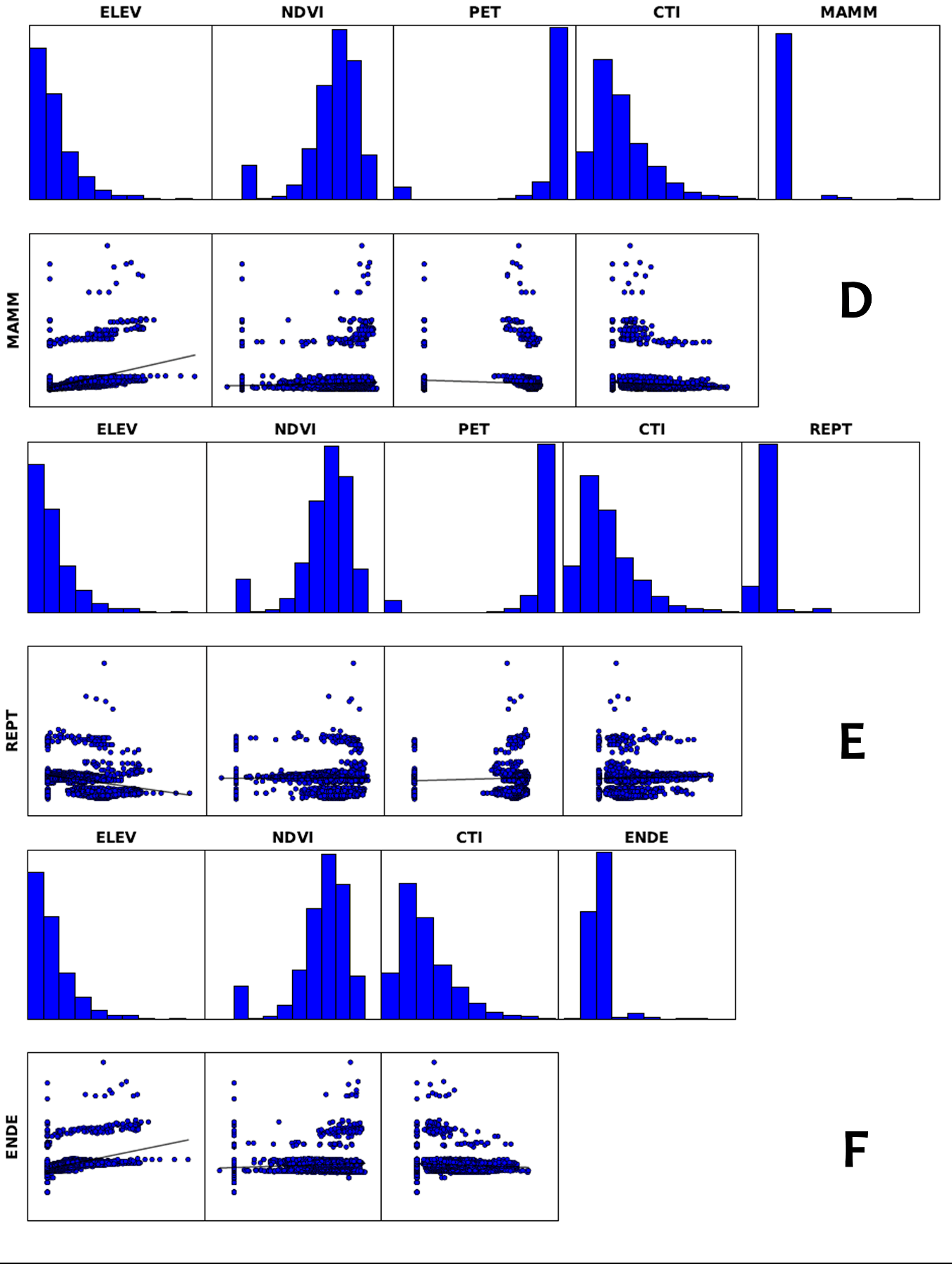


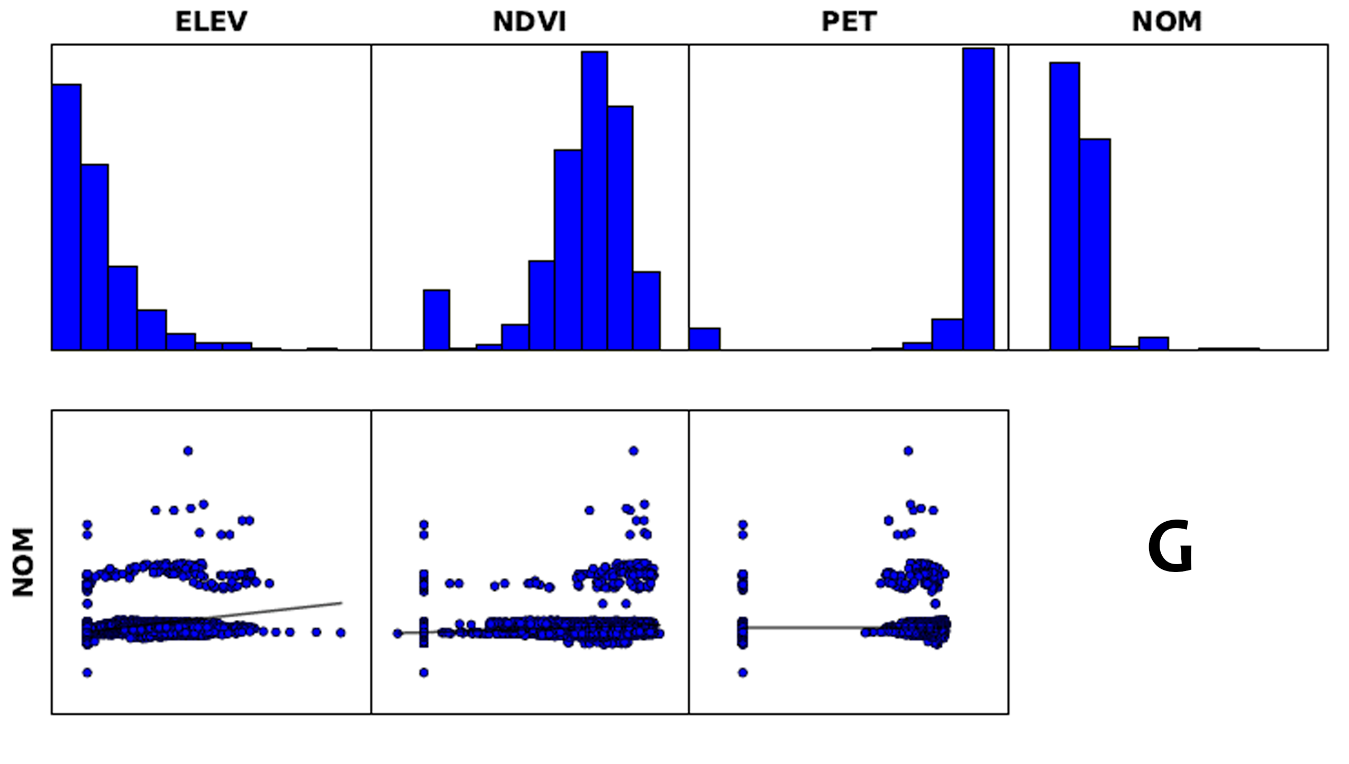

Supplement: S3 File — (DOCX) [file pone.0267589.s005.docx]
